# Large strain correction for tunnel analyses considering hydromechanical coupling and ground anisotropy

**DOI:** 10.1038/s41598-023-42158-2

**Published:** 2023-09-26

**Authors:** Matteo Natale, Alexandros N. Nordas, Seraina Kopp, Georgios Anagnostou

**Affiliations:** ETH Zurich, Stefano-Franscini-Platz 5, 8093 Zurich, Switzerland

**Keywords:** Engineering, Civil engineering

## Abstract

The deformations resulting from tunnel analyses for heavily squeezing ground may be very large, necessitating numerical formulations that consider geometric nonlinearity. Alternatively, for a certain class of problems, routine small strain analyses can be performed, and their results can be corrected to account for large strains by means of a simple hyperbolic expression proposed a few years ago. The present paper shows that this correction equation is sufficiently accurate for practical purposes even in the case of anisotropic material behaviour and for hydromechanically coupled, steady state or transient analyses of tunnels. The accuracy of the equation prediction varies amongst these cases but is satisfactory overall for the purpose of preliminary calculations, thus broadening its value and usefulness as a preliminary design tool.

## Introduction

In the analysis of tunnelling through heavily squeezing rock, where deformations can be very large, the application of small-strain theory, which evaluates stiffness and equilibrium in the undeformed initial configuration, can significantly overestimate tunnel convergences and lead even to nonsensical predictions. Such problems must be solved instead in the framework of the large-strain theory which considers geometric nonlinearity by evaluating stiffness and equilibrium in the deformed configuration.

Vrakas and Anagnostou^1^ showed that the differences between small- and large-strain analyses become considerable when convergences exceed 20% of the tunnel radius, demonstrating the limitations of small-strain analyses on prominent examples of tunnelling under heavily squeezing conditions: In a critical 6 km stretch of the Gotthard base tunnel in Switzerland, which crosses low stiffness and strength kakiritic rocks at depths up to 900 m, small-strain analysis was shown to overestimate the over-excavation required to accommodate the designated profile clearance by more than 50%. In the Yacambú-Quibor tunnel in Venezuela, where complete closure of the cross-section (convergence > 80%) occurred in a 23.3 km stretch crossing weak graphitic phyllites at depths up to 1270 m, a back-calculation based on small-strain analysis was shown to result in a fourfold overestimate of the ground cohesion. In a critical zone of the planned subaqueous Gibraltar Strait tunnel, which crosses breccia over 4 km at a depth of 200 m below the seabed, preliminary small-strain calculations of the undrained ground response were shown to yield physically unrealistic predictions (convergences > 100% vs. 48% from large-strain analyses).

While the above examples underscore the significance and necessity of adopting a large-strain formulation in the analysis of severe squeezing conditions, geometrically nonlinear finite element (FE) analyses can be excessively demanding in respect of computing time and resources. In view of this, Vrakas and Anagnostou^1^ proposed the following simple, accurate and theoretically well-founded hyperbolic equation for obtaining large-strain solutions from small-strain analyses:

1 $$U_{a,ls} = 1 - \frac{1}{{\sqrt {1 + 2U_{a,ss} } }},$$

where *U*_*a,ss*_ = *u*_*a,ss*_/*a* and *U*_*a,ls*_ = *u*_*a,ls*_/*a*, *u*_*a,ss*_ and *u*_*a,ls*_ are the displacements from small- and large-strain analyses, respectively, and *a* denotes the tunnel radius in the initial undeformed configuration. Equation (1) constitutes a valuable tool for preliminary design, since it entails performance only of routine small-strain analyses with a subsequent “self-correction” of the results. Although it has been derived considering the plane-strain and rotationally symmetric tunnel problem, it has been shown to be reasonably accurate also for other, general 2D problems (cylindrical and spherical openings, dilatant and hardening material behaviours, non-cylindrical tunnels and non-hydrostatic *in-situ* stress fields, undrained response of saturated low-permeability ground), as well as for 3D problems of an advancing tunnel heading.

Building upon the work of Vrakas and Anagnostou^1^, the present paper investigates the applicability of Eq. (1) (hereafter referred also as “correction equation”) to anisotropic problems (Section Anisotropic stress analyses), and to hydromechanically coupled, steady state or transient problems with isotropic (Section Coupled isotropic analyses) or anisotropic (Section Coupled anisotropic analyses) material, which have not been considered previously. Throughout the paper, either isotropic or transversely isotropic rocks with a vertical axis of symmetry and horizontal planes of anisotropy (foliation, stratification) are considered. The constitutive behaviour of the rock is assumed as in Vrakas and Anagnostou^1^, *i.e.* linear-elastic and perfectly plastic, following the Mohr–Coulomb yield criterion and a non-associated flow rule, which is the most commonly used model in rock and tunnel engineering practice.

## Anisotropic stress analyses

### Problem layout and computational assumptions

**Figure 1:**
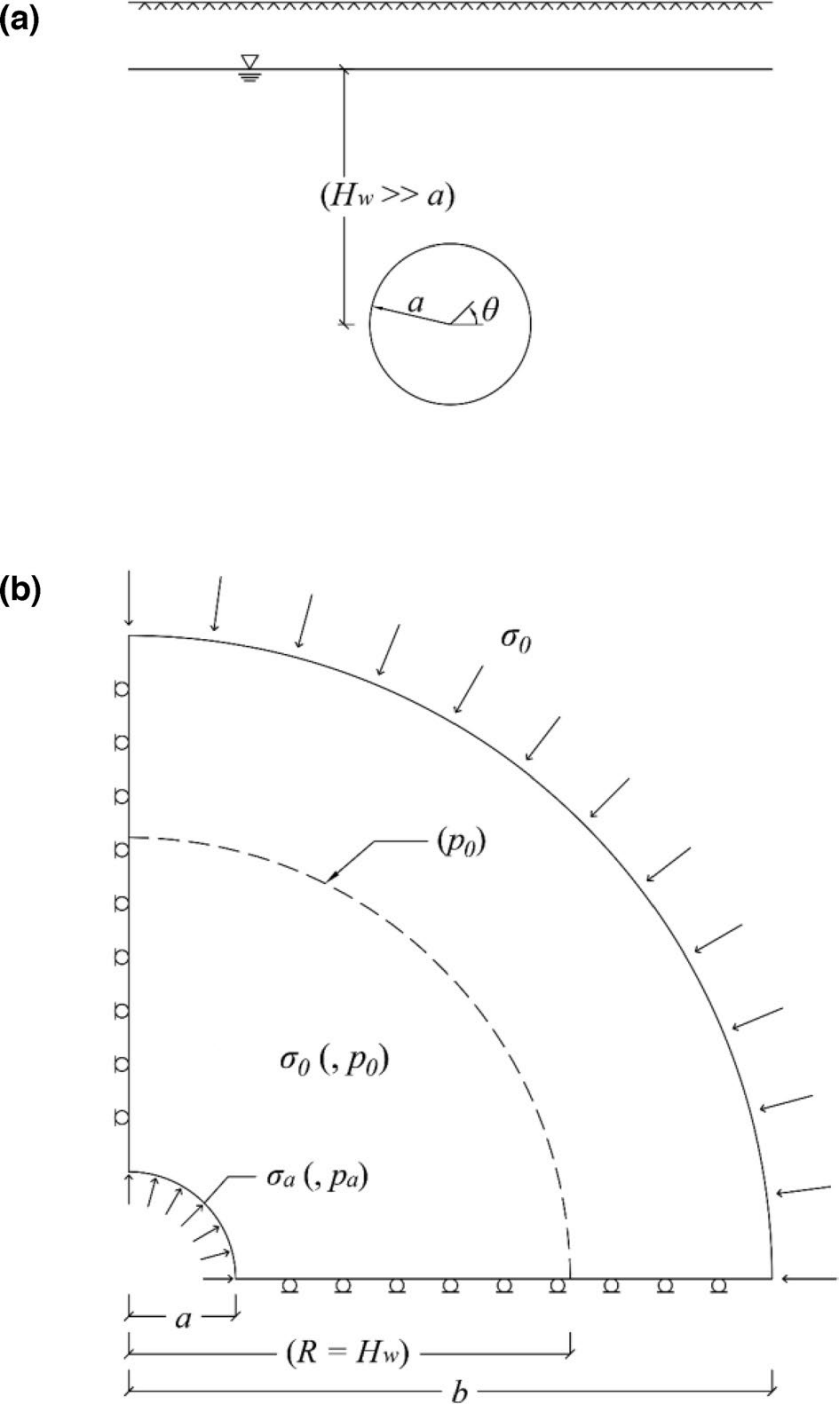
(**a**) Problem layout and, (**b**), model (data in brackets: applies to the coupled analyses only).

Consideration is given herein to the plane-strain problem of a deep, cylindrical tunnel of radius *a*, crossing homogeneous, transversally isotropic rock and subjected to a homogeneous and hydrostatic *in-situ* stress field (Fig. 1). The computational domain considers one quarter of the tunnel cross-section, on account of symmetry about the horizontal and vertical planes, and a far field boundary (where the radial *in-situ* stress *σ*_*0*_ prevailing at the elevation of the tunnel axis is applied) at a sufficiently large distance from the tunnel (*b* = 100 *a*; Fig. 1b). The ground response to tunnelling is simulated via an unloading of the tunnel boundary from the initial *in-situ* stress *σ*_*0*_ to zero, which provides the maximum convergence. The numerical computations have been performed in Abaqus®^2^, employing a structured FE mesh of 1′120 4-noded, linear, quadrilateral, plane strain elements.

A linear elastic and perfectly plastic constitutive model with a Mohr–Coulomb yield criterion and a non-associated flow rule is adopted, which considers transversal isotropy and reduced strength for shearing along an anisotropy plane compared to shearing through the rock matrix. The model behaviour is defined by 11 constants^3^: two Young’s moduli *E*_*p*_ and *E*_*o*_ ≤ *E*_*p*_ parallel and orthogonal to the anisotropy plane, respectively; two Poisson’s ratios of the anisotropy plane *ν*_*pp*_, *ν*_*op*_ for loading parallel and orthogonal to it, respectively; the cohesion *c*, angle of internal friction *φ* and angle of dilation *ψ* for shearing through the matrix; the three corresponding plasticity constants (*c*_*b*_,* φ*_*b*_,* ψ*_*b*_) for shearing along an anisotropy plane; and the shear modulus *G*_*op*_ on planes orthogonal to the anisotropy plane. The latter is an independent material constant of the transversely isotropic material and is taken equal to 0.5 *E*_*o*_/(1 + *ν*_*op*_), as suggested by Wittke^4^.

### Accuracy of the correction equation

Dimensional analysis makes it possible to express the normalised displacement of any point of the tunnel boundary in the following form:

2 $$U_{a} = \frac{{u_{a} }}{a} = f\left( {\frac{{E_{p} }}{{\sigma_{0} }},\;\frac{{E_{p} }}{{E_{o} }},\;\nu_{pp} ,\;\nu_{op} ,\varphi ,\;\frac{{f_{c} }}{{\sigma_{0} }},\psi ,\;\frac{{\varphi_{b} }}{\varphi },\;\;\frac{{c_{b} }}{c},\psi_{b} ,\;\theta \;} \right),$$

where *f*_*c*_ is the uniaxial compressive strength disregarding strength anisotropy (*f*_*c*_ = 2 *c* cos*φ* /(1—sin*φ*)), and *θ* denotes the angular position of a point on the tunnel boundary. The parameter sets considered are given in Table 1 and have been selected such that the normalised convergences from small-strain analyses (*U*_*a,ss*_) range between 15 and 100%. The sets with *c*_*b*_/*c* = ∞ disregard strength anisotropy and are intended to consider the isolated effect of stiffness anisotropy.

**Table 1:** Parameter sets of the anisotropic stress analyses (parameters common to all sets: *ν*_*pp*_ = *ν*_*op*_ = 0.2, *φ* = 25°, *ψ* = *ψ*_*b*_ = 5°).

Set	*E*_*p*_/*σ*_*o*_	*f*_*c*_/*σ*_*o*_	*E*_*p*_/*E*_*o*_	*c*_*b*_/*c*	*φ*_*b*_/*φ*
1	100.0	0.21	1.5	∞	1
2	100.0	0.21	3	∞	1
3	100.0	0.21	1.5	0.25	1
4	100.0	0.21	3	0.25	1
5	100.0	0.21	1.5	1	0.6
6	100.0	0.21	3	1	0.6
7	100.0	0.21	1.5	0.25	0.6
8	100.0	0.21	3	0.25	0.6
9	54.5	0.11	1.5	∞	1
10	54.5	0.11	3	∞	1
11	54.5	0.11	1.5	0.25	1
12	54.5	0.11	3	0.25	1
13	54.5	0.11	1.5	1	0.6
14	54.5	0.11	3	1	0.6
15	54.5	0.11	1.5	0.25	0.6
16	35.3	0.07	1.5	∞	1
17	35.3	0.07	3	∞	1
18	35.3	0.07	1.5	0.25	1
19	35.3	0.07	3	0.25	1
20	35.3	0.07	1.5	1	0.6

**Figure 2:**
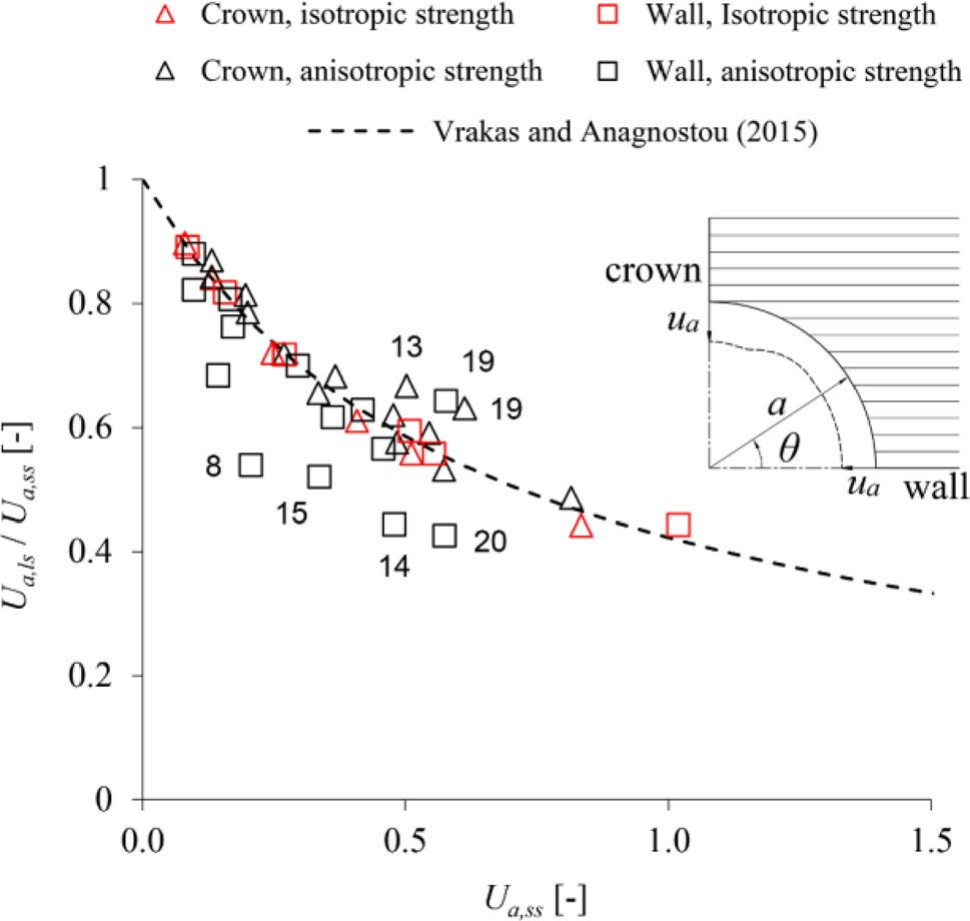
Anisotropic stress analyses: ratio of the large- to the small-strain displacements versus the small-strain displacements (parameters: s. Table 1; labels besides the markers: data-set numbers after Table 1).

Anisotropy results in general in a non-uniform deformation of the tunnel boundary. For the assumed horizontal orientation of the anisotropy planes, stiffness anisotropy results in higher convergences at the crown (*θ* = 90°), where unloading takes place in the “softer” direction orthogonal to the planes, compared to the sidewall (*θ* = 0°), where unloading takes place in the “stiffer” direction parallel to the planes (see inset in Fig. 2). Strength anisotropy results in a local increase of convergences over a narrow region close to the crown, where the lower strength of shearing along the anisotropy planes prevails; however, this effect is in general small in comparison with the absolute magnitude of convergence considered. The crown and sidewall can thus be considered as reference points for evaluating the results, since their convergences are indicative of the maximum and minimum over the boundary, and their average value is also approximately equal to the average convergence over the entire boundary.

Figure 2 shows the numerically determined ratio *U*_*a,ls*_/*U*_*a,ss*_ as a function of the predictions *U*_*a,ss*_ of small-strain analyses at the tunnel sidewall (triangular markers) and crown (square markers), along with the approximation of this relationship based on Eq. (1) (dashed line). One can readily verify that the correction equation approximates the numerical predictions with very high accuracy in the absence of strength anisotropy (red markers). In cases considering strength anisotropy (black markers), the correction equation is less accurate overall, mostly slightly underestimating the convergences at the crown and overestimating those at the sidewall; however, it still provides reasonably accurate prediction of the average convergence throughout the considered range, even for excessive convergences *U*_*a,ss*_ close to 100%.

## Coupled isotropic analyses

### Problem layout and computational assumptions

Consideration is given in the sequel to a tunnel deep under the water table (*H*_*w*_ = 100 *a*; Fig. 1a). The seepage flow field is approximated as rotationally symmetric, assuming that the pore pressure is equal to the *in-situ* hydrostatic pressure *p*_*0*_ prevailing at the elevation of the tunnel axis at a radius *R* = *H*_*w*_ (Fig. 1b); this simplification has been shown to be sufficiently accurate^5, 6^. Identical computational specifications to those outlined in Section Anisotropic stress analyses are otherwise adopted, whilst also considering the linear interpolation of the pore pressure within each FE.

The rock is considered as a porous, fully saturated medium obeying Terzaghi’s principle of effective stresses and isotropic Darcy’s law. Two borderline cases are investigated for the permeability: a high permeability (*k* = 10^–6^ m/s) where drained conditions prevail continuously; and a low permeability (*k* = 10^–13^ m/s) where the conditions are undrained during tunnel excavation, and become drained after a sufficiently long transient consolidation period. The parameters adopted in the computations are given in Table 2.

**Table 2:** Parameter sets of the isotropic coupled analyses (parameters common to all sets: *ν* = 0.3, *ψ* = 0°, *p*_*0*_/*σ*_*0*_ = 0.4, *b*/*a* = *R*/*a* = 100).

Set	*E*/*σ*_*o*_	*f*_*c*_/*σ*_*o*_	*φ* [°]
1	160	0.16	20
2	80	0.23	20
3	40	0.22	30
4	20	0.28	30
5	160	0.13	20
6	80	0.15	20
7	40	0.12	30
8	20	0.15	30
9	160	0.11	20
10	80	0.12	20
11	40	0.10	30
12	20	0.12	30

The high-permeability case assumes that the hydraulic head field readily achieves steady state prior to excavation, as in the case of perfect advance drainage^7^. This is achieved by initially prescribing atmospheric pore pressure over the fixed tunnel boundary (*p*_*a*_ = 0, where *p*_*a*_ denotes the pore pressure at the tunnel boundary). Subsequently, the boundary is completely unloaded while maintaining the atmospheric pressure boundary condition.

**Figure 3:**
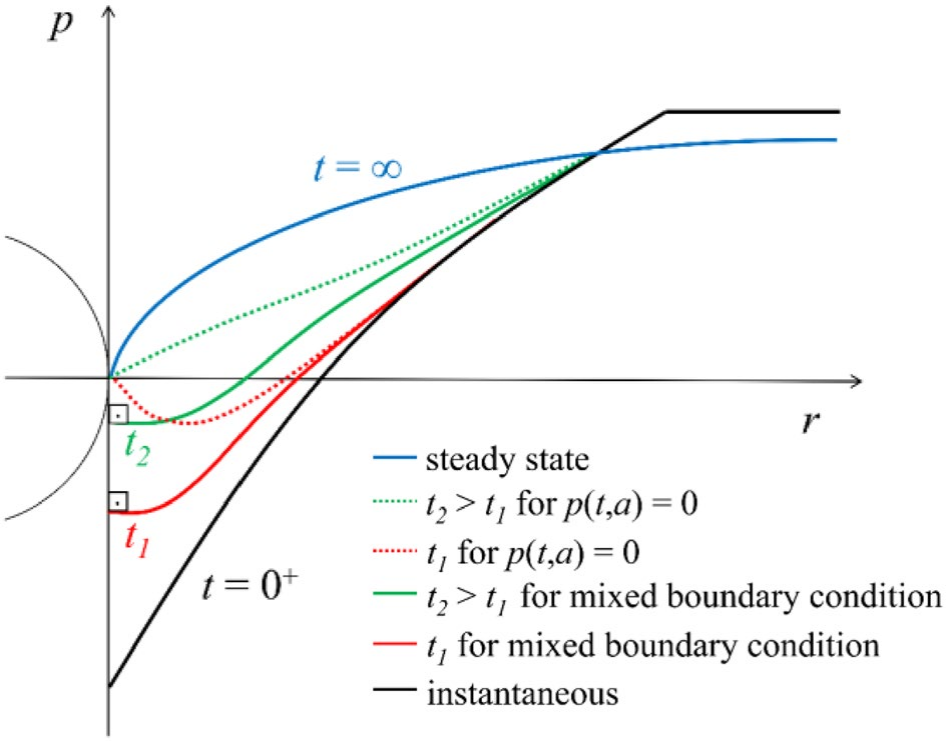
Typical radial pore pressure distributions.

The low-permeability case initially considers a complete unloading of the tunnel boundary assuming a no-flow hydraulic boundary condition (*q*_*a*_ = 0, where *q*_*a*_ denotes the flux at the tunnel boundary), which provides the instantaneous undrained convergence. Subsequently, a transient consolidation analysis is performed until steady state is achieved, considering a mixed hydraulic boundary condition over the tunnel boundary. This ensures water seepage from the ground into the tunnel, but not vice versa (*q*_*a*_ = 0 if *∂p/∂r* < 0, *p*_*a*_ = 0 if *∂p/∂r* > 0);^8^, if an atmospheric pressure condition (*p*_*a*_ = 0) was specified instead, watering of the ground from the tunnel could occur in the early stages of consolidation, when negative pore pressures may develop. The effect of the adopted hydraulic boundary condition is illustrated qualitatively for two time-instances (*t*_1_, *t*_2_) in Fig. 3.

### Accuracy of the correction equation

Dimensional analysis enables expression of the normalised convergence of the tunnel boundary in the following form:

3 $$U_{a} = \frac{{u_{a} }}{a} = f\left( {\frac{E}{{\sigma_{0} }},\;\;\nu ,\;\varphi ,\;\frac{{f_{c} }}{{\sigma_{0} }},\psi ,\;\frac{{p_{0} }}{{\sigma_{0} }}\;} \right)$$

The parameters adopted in the computations are given in Table 2.

**Figure 4:**
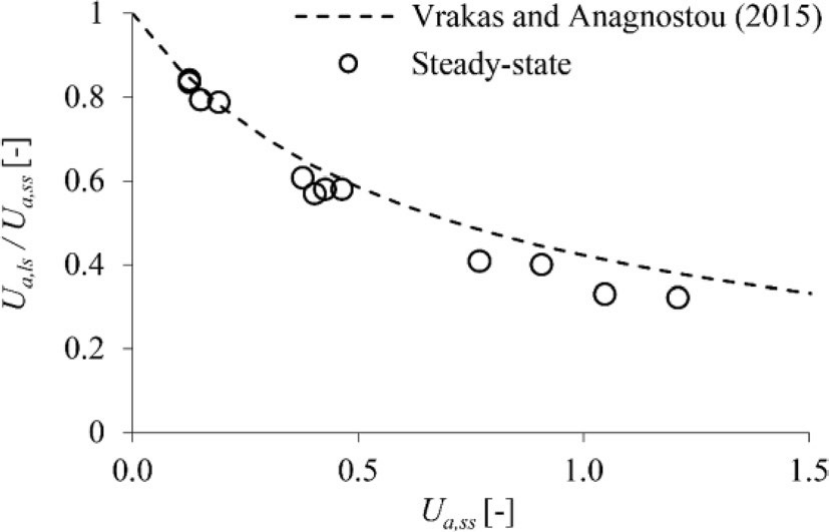
Isotropic coupled analyses for high-permeability ground: ratio of the large- to the small-strain displacements versus the small-strain displacements (parameters: s. Table 2).

Figure 4 shows the numerically determined ratio *U*_*a,ls*_/*U*_*a,ss*_ as a function of the predictions *U*_*a,ss*_ of small-strain analyses (round markers) for the high-permeability case, along with the approximation of this relationship based on Eq. (1) (dashed line). The correction equation achieves a very good agreement with the numerical predictions, becoming only slightly conservative towards the range of excessively large *U*_*a,ss*_.

**Figure 5:**
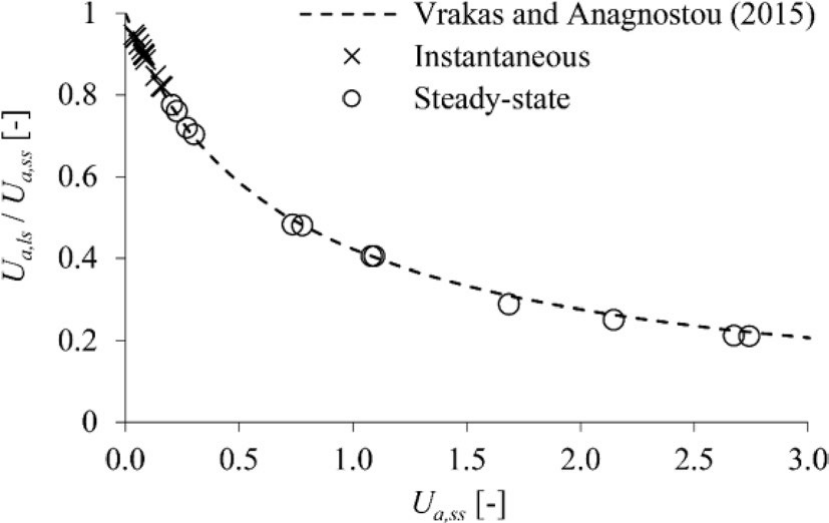
Isotropic coupled analyses for low-permeability ground: ratio of the large- to the small-strain displacements versus the small-strain displacements (parameters: s. Table 2).

The results for the case of low permeability are shown in Fig. 5, where it is readily seen that the correction equation achieves a virtually perfect match with the numerical predictions. This is expected for the instantaneous convergences, since these lie in the range where geometric nonlinearity effects are limited (*U*_*a,ss*_ < 15%), but holds also for the steady-state convergences, even in the range of excessively large, nonsensical values (*U*_*a,ss*_ > 100%).

## Coupled anisotropic analyses

The applicability of the correction equation to coupled anisotropic problems is examined considering the computational model discussed in Section Coupled isotropic analyses, with the additional specification of an anisotropic material behaviour analogously to Section Anisotropic stress analyses. The parameters adopted in the computations are given in Tables 3 and 4 for the high- and low-permeability cases, respectively. In addition to the strength and stiffness anisotropy, the case of a pronouncedly anisotropic seepage flow was considered in certain cases, assuming that the permeability parallel to the bedding or schistosity plane is 10 times higher than perpendicularly to it.

**Table 3:** Parameter sets of the anisotropic coupled analyses for high-permeability ground (parameters common to all sets: *ν*_*pp*_ = *ν*_*op*_ = 0.2, *φ* = 25°, *ψ* = *ψ*_*b*_ = 5°, *b*/*a* = *R*/*a* = 100).

Set	*E*_*p*_/*σ*_*o*_	*f*_*c*_/*σ*_*o*_	*E*_*p*_/*E*_*o*_	*c*_*b*_/*c*	*φ*_*b*_/*φ*	*p*_*0*_/*σ*_*0*_
1	100.0	0.21	1.5	∞	1	0.23
2	100.0	0.21	3	∞	1	0.23
3	100.0	0.21	1.5	0.25	1	0.23
4^(a)^	100.0	0.21	3	0.25	1	0.23
5	100.0	0.21	1.5	1	0.6	0.23
6	100.0	0.21	3	1	0.6	0.23
7	100.0	0.21	1.5	0.25	0.6	0.23
8	100.0	0.21	3	0.25	0.6	0.23
9	54.5	0.11	1.5	∞	1	0.13
10	54.5	0.11	3	∞	1	0.13
11	54.5	0.11	1.5	0.25	1	0.13
12^(a)^	54.5	0.11	3	0.25	1	0.13
13	54.5	0.11	1.5	1	0.6	0.13
14^(a)^	54.5	0.11	3	1	0.6	0.13
15	54.5	0.11	1.5	0.25	0.6	0.13
16	40.0	0.08	1.5	∞	1	0.09
17^(a)^	35.3	0.08	3	∞	1	0.09
18^a^	35.3	0.08	1.5	0.25	1	0.09
19	35.3	0.08	3	0.25	1	0.09

^a^For this data-set the case of permeability anisotropy with *k*_*h*_/*k*_*v*_ = 10 has been additionally considered.

**Table 4:** Parameter sets of the anisotropic coupled analyses for low-permeability ground (parameters common to all sets: *ν*_*pp*_ = *ν*_*op*_ = 0.2, *φ* = 25°, *ψ* = *ψ*_*b*_ = 5°, *b*/*a* = *R*/*a* = 100).

Set	*E*_*p*_/*σ*_*o*_	*f*_*c*_/*σ*_*o*_	*E*_*p*_/*E*_*o*_	*c*_*b*_/*c*	*φ*_*b*_/*φ*	*p*_*0*_/*σ*_*0*_
1	100.0	0.21	1.5	∞	1	0.23
2	100.0	0.21	3	∞	1	0.23
3^a^	100.0	0.21	1.5	0.25	1	0.23
4^a^	100.0	0.21	3	0.25	1	0.23
5	100.0	0.21	1.5	1	0.6	0.23
6	100.0	0.21	3	1	0.6	0.23
7	100.0	0.21	1.5	0.25	0.6	0.23
8^a^	100.0	0.21	3	0.25	0.6	0.23
9	66.7	0.14	1.5	∞	1	0.16
10	66.7	0.14	3	∞	1	0.16
11^a^	66.7	0.14	1.5	0.25	1	0.16
12^a^	66.7	0.14	3	0.25	1	0.16
13	66.7	0.14	1.5	1	0.6	0.16
14	66.7	0.14	3	1	0.6	0.16
15^a^	66.7	0.14	1.5	0.25	0.6	0.16
16^a^	66.7	0.14	3	0.25	1	0.16
17	50.0	0.10	1.5	∞	1	0.12
18	50.0	0.10	3	∞	1	0.12
19	50.0	0.10	1.5	0.25	1	0.12
20	50.0	0.10	3	0.25	1	0.12
21	50.0	0.10	1.5	1	0.6	0.12
22^a^	50.0	0.10	1.5	0.25	0.6	0.12

^a^For this data-set the case of permeability anisotropy with *k*_*h*_/*k*_*v*_ = 10 has been additionally considered.

**Figure 6:**
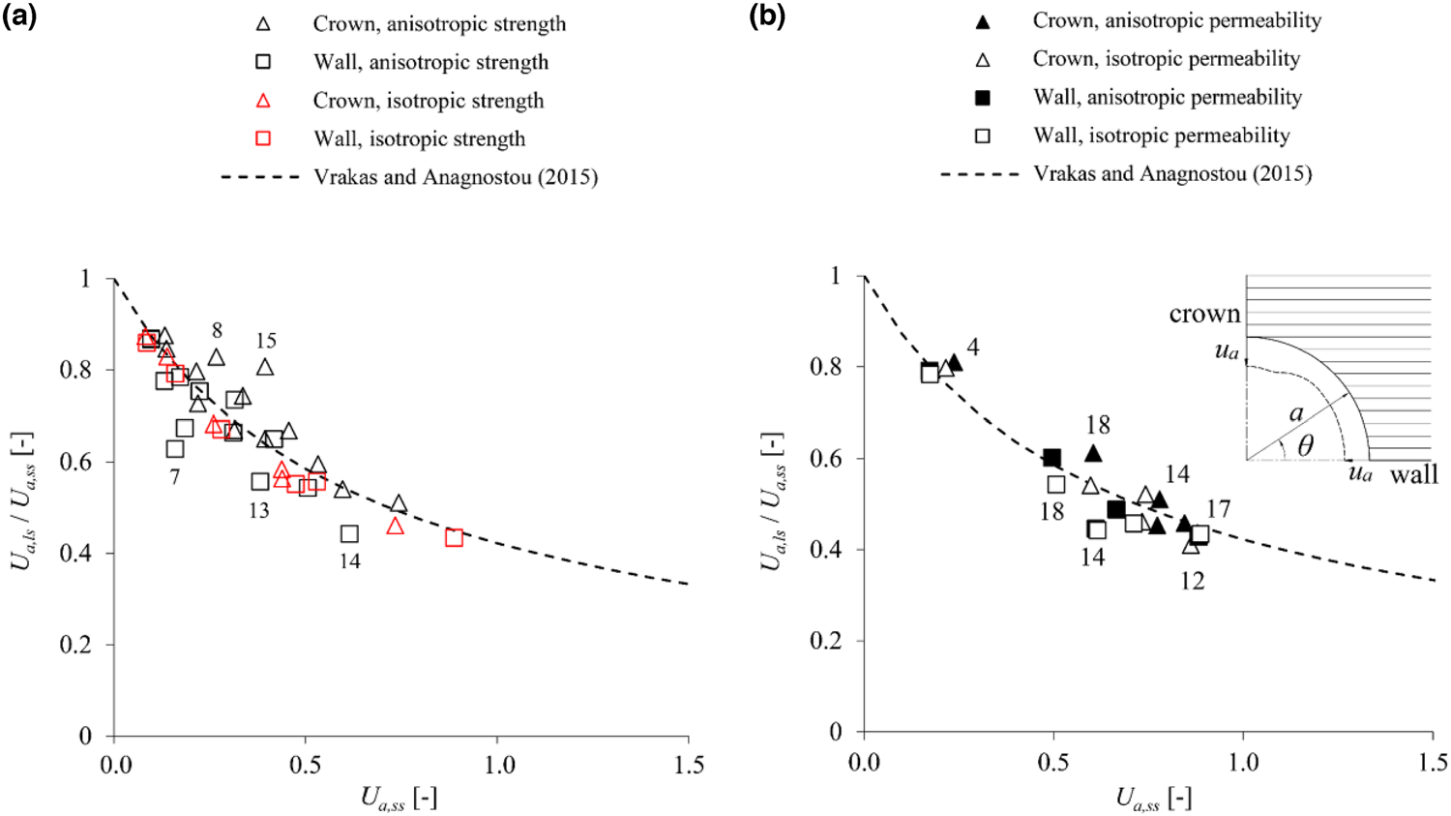
Anisotropic coupled analyses for high-permeability ground: ratio of the large- to the small-strain displacements versus the small-strain displacements assuming, (**a**) isotropic permeability or, (**b**) isotropic and anisotropic permeability (parameters: s. Table 3; labels besides the markers: data-set numbers after Table 3).

Figure 6a assumes isotropic permeability and shows the numerically determined ratio *U*_*a,ls*_/*U*_*a,ss*_ as a function of the predictions *U*_*a,ss*_ of small-strain analyses, along with the approximation of this relationship based on Eq. (1) (dashed line) for the high-permeability case. The correction equation is very accurate in the absence of strength anisotropy (red markers) and reasonably accurate otherwise (black markers), slightly underestimating the displacements at the crown and overestimating those at the sidewall in some cases; overall, the accuracy achieved is very good in terms of average convergences. This also applies in the case of anisotropic permeability (Fig. 6b).

**Figure 7:**
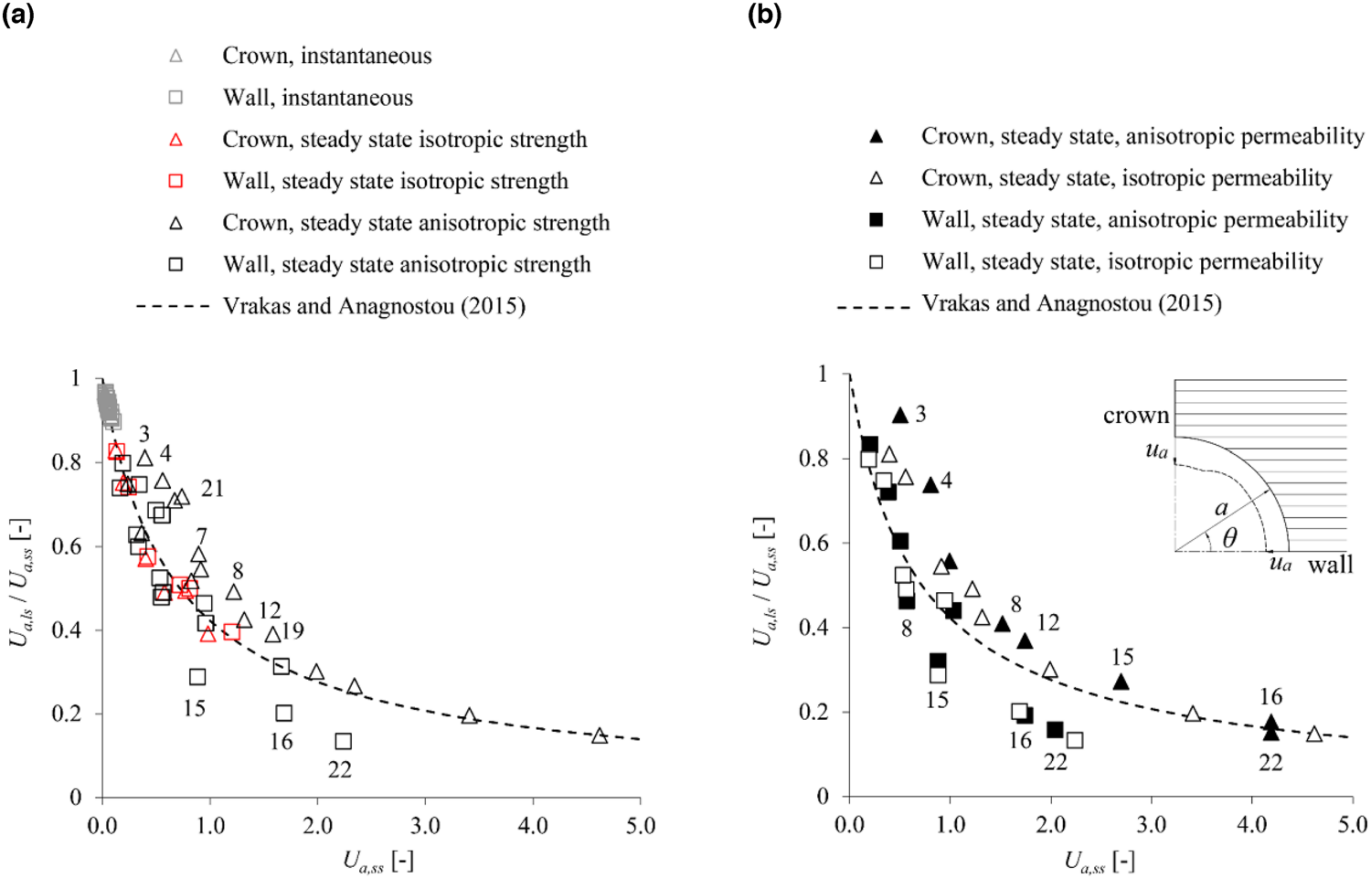
Anisotropic coupled analyses for low-permeability ground: ratio of the large- to the small-strain displacements versus the small-strain displacements assuming, (**a**) isotropic permeability or (**b**) isotropic and anisotropic permeability (parameters: s. Table 3; labels besides the markers: data-set numbers after Table 4).

Figure 7a shows the results for the low-permeability case, considering isotropic permeability. The instantaneous (undrained) convergences *U*_*a,ss*_ are anyway small (< 15%) and thus accurately predicted even by the infinitesimal-strain analysis. As for the steady-state convergences, the accuracy of the correction equation is high in the absence of strength anisotropy (red markers) but inferior otherwise (black markers), particularly for the crown, where the correction equation may underestimate displacement by up to 40% (data set 21). There are also some outliers (tunnel wall; data sets 15, 16 and 22) for which even the correction equation overestimates displacement. Notwithstanding the inaccuracies observed in the case of strength anisotropy, the correction equation provides a reasonable indication of the anticipated magnitude of convergences; therefore, it is still valuable for preliminary calculation purposes, particularly in cases where small-strain analyses provide unusable results, *i.e.* predicting excessive and nonsensical convergences (*U*_*a,ss*_ > 100%). The same conclusions can be drawn when considering permeability anisotropy (Fig. 7b).

## Concluding remarks

By utilising parametric plane strain numerical simulations, the present paper demonstrated that the equation proposed by Vrakas and Anagnostou^1^ for obtaining large-strain solutions from small-strain analyses can be applied also to anisotropic and hydromechanically coupled, steady state or transient analyses. The accuracy of the equation has been shown to be: (i) sufficient for estimating average convergences in anisotropic ground (Fig. 2); (ii) very high for estimating instantaneous undrained convergences in low-permeability isotropic (Fig. 5) or anisotropic (Fig. 7) ground; (iii) very high for estimating steady state convergences in high-permeability (Fig. 4) and low-permeability (Fig. 5) isotropic ground; (iv) reasonable for estimating steady state convergences in high-permeability (Fig. 6) and low-permeability (Fig. 7) anisotropic ground. Complementing the work of Vrakas and Anagnostou^1^, the present findings broaden even further the already wide applicability of the correction equation as a preliminary design tool.

## List of symbols

*E*_*o*_: Young’s modulus normal to the anisotropy plane
*E*_*p*_: Young’s modulus parallel to the anisotropy plane
*G*_*op*_: Shear modulus on planes orthogonal to the anisotropy plane
*H*_*w*_: Depth of the tunnel underneath the water table
*R*: Outer radius of the region affected by tunnel drainage
*U*_*a,ss*_: Normalised small-strain radial displacement of the tunnel boundary
*U*_*a,ls*_: Normalised large-strain radial displacement of the tunnel boundary
*a*: Tunnel radius
*b*: Radius of the far field boundary
*c*: Cohesion
*c*_*b*_: Cohesion in the anisotropy plane
*f*_*c*_: Uniaxial compressive strength disregarding strength anisotropy
*k*: Permeability
*k*_*h*_: Permeability in the horizontal plane
*k*_*v*_: Permeability in the vertical direction
*p*: Pore pressure field
*p*_*0*_: *In-situ* pore pressure at the elevation of the tunnel axis
*p*_*a*_: Pore pressure at the tunnel boundary
*q*_*a*_: Flux at the tunnel boundary
*r*: Radial coordinate
*t*: Time
*u*_*a*_: Radial displacement of the tunnel boundary
*u*_*a,sss*_: Small-strain radial displacement of the tunnel boundary
*u*_*a,ls*_: Large-strain radial displacement of the tunnel boundary
*θ*: Angular coordinate
*φ*: Angle of internal friction
*φ*_*b*_: Angle of internal friction in the anisotropy plane
*ν*_*op*_: Poisson ratio expressing the effect of the normal stresses orthogonal to the anisotropy plane to the normal strains parallel to the anisotropy plane
*ν*_*pp*_: Poisson ratio expressing effect of the normal stresses parallel to the anisotropy plane to the normal strains parallel to the anisotropy plane
*σ*_0_: *In-situ* stress at the elevation of the tunnel axis
*σ*_*a*_: Radial support pressure at the tunnel boundary
*ψ*: Dilatancy angle
*ψ*_*b*_: Dilatancy angle in the anisotropy plane
